# An Unusual Piceatannol Dimer from *Rheum austral* D. Don with Antioxidant Activity

**DOI:** 10.3390/molecules190811453

**Published:** 2014-08-04

**Authors:** Lin Hu, Na-Na Chen, Qun Hu, Cui Yang, Qing-Song Yang, Fang-Fang Wang

**Affiliations:** 1 Key Laboratory of Chemistry in Ethnic Medicinal Resources, State Ethnic Affairs Commission & Ministry of Education, Yunnan University of Nationalities, Kunming 650031, China; E-Mails: cnana1111@126.com (N.-N.C.); yangynni@163.com (C.Y.); smkms@126.com (Q.-S.Y.); foreverfang98@126.com (F.-F.W.); 2 Kunming Xianghao Technology Co., Ltd., Kunming 650204, China; E-Mail: huqun871@163.com

**Keywords:** *Rheum austral* D. Don, piceatannol dimer, rheumaustralin, DPPH radical

## Abstract

A novel dimer of piceatannol glycoside, named rheumaustralin (**1**) was isolated from the underground parts of the ethnomedicinal plant *Rheum austral* (Polygonaceae) collected from Tibet together with 17 known compounds, including rheumin (**2**), 2,5-dimethyl-7-hydroxychromone (**3**), 2,5-dimethylchromone-7-*O*-β-d-glucopyranoside (**4**), 7-hydroxy-2-(2'-hydroxypropyl)-5-methylchromone (**5**), torachrysone (**6**) torachrysone-8-*O*-β-d-glucopyranoside (**7**), 4-(4'-hydroxyphenyl)-2-butanone-4'-*O*-β-d-glucopyranoside (**8**), amabiloside (**9**), *N*-*trans*-feruloyl tyramine (**10**), chrysophanol (**11**), aloe-emodin (**12**), emodin (1**3**), physcion (**14**), physcion-1-*O*-β-d-glucopyranoside (**15**), emodin-8-*O*-β-d-glucopyranoside (**16)**, d-catechin (**17**) and gallic acid (**18**). Their structures were determined by combined spectroscopic methods and by comparison of their spectral data with those reported in literature. Compounds **1**–**10** were tested for their ability to scavenge 1, 1-diphenyl-2-picrylhydrazyl (DPPH) radical.

## 1. Introduction

*Rheum* *australe* D. Don (syn. *Rheum emodi* Wall. ex Meissn., Polygonaceae) is a robust, perennial herb with stout rhizomes. The distribution of this plant is confined to the Himalayan region, covering the areas of India (Kashmir and Sikkim), Bhutan, Nepal, Pakistan, Myanmar, and China [1]. The roots of *R.* *australe* are widely used in Ayurvedic and Chinese folk medicine as a purgative, stomachic, astringent and tonic and for piles, chronic bronchitis and asthma, as well as in certain skin diseases. *R.* *australe* plants produce diverse phenolic metabolites. More than 56 compounds, belonging to anthraquinones, stilbenes, anthrones, oxanthrone ethers and esters, chromones, flavonoids, carbohydrates, lignans, phenols, and sterols have been identified or characterized from the roots and rhizomes of this plant collected from Nepal, India, Czech Republic (cultivated), and China [2,3,4]. Previous investigations on the constituents of *R.* *australe* collected from Tibet, locally known as “Zang Bian Dahuang”, led to the isolation of a series of piceatannol glycosides and anthraquinones as its major components [5,6]. Some of these compounds demonstrated a wide range of biological and pharmacological properties such as antioxidant [7,8], antifungal [9], cytotoxic [10], hypoglycemic [11], anti-tuberculosis [12], neuron protective [13] and antiviral [14] activities. As a part of a program to study the antioxidant secondary metabolits of *Rheum* plants from the Qinghai-Tibetan region of China [15], a novel dimer of piceatannol-4'-*O*-β-d-glucopyranoside, named rheumaustralin (1), was obtained from the underground parts of this ethnomedicinal plant, together with 17 phenolic compounds including pyranones, naphthalenes, chromones, anthraquinones, flavonoids, phenolic amides and some simple aromatic compounds. Herein, we describe the isolation, structural elucidation of these compounds, as well as the DPPH free radical scavenging activities of compounds **1**–**10**.

## 2. Results and Discussion

### 2.1. Structural Elucidation of the New Compound

**Figure 1:**
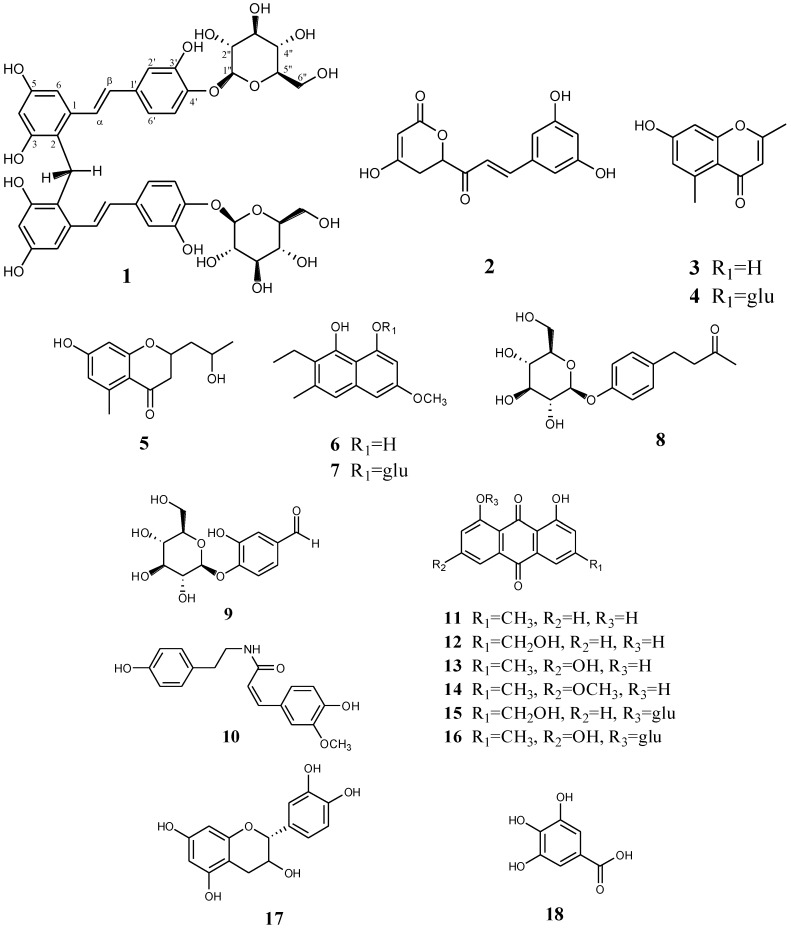
Chemical structures of compounds **1**–**18**.

The isolated compounds were identified by different spectroscopic analyses, including the extensive use of HR-ESI-MS, 1D (^1^H and ^13^C) and 2D-NMR techniques (HSQC, HMBC), and by comparing the experimental NMR data to values reported in the literature. The structures of the isolated compounds are shown in Figure 1.

**Figure 2:**
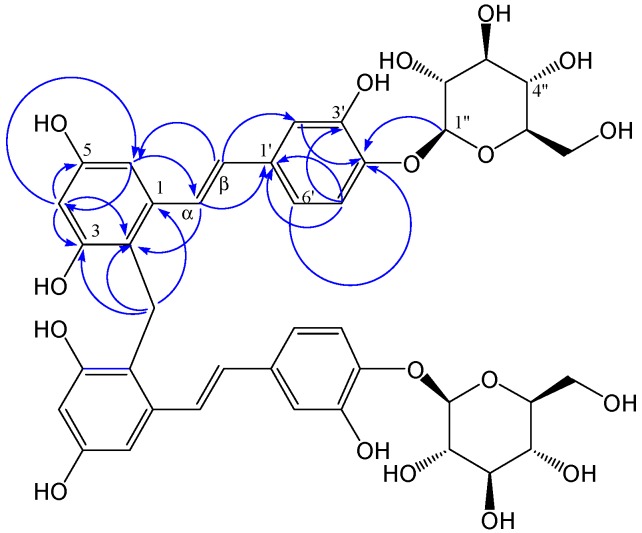
Main HMBC (indicated by blue arrows from ^1^H to ^13^C) of compound **1**.

Compound **1** was obtained as a yellow powder with an optical rotation value 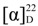 +50.3030 (c = 0.22, MeOH). The molecular formula of compound **1**, C_41_H_44_O_18_, was deduced from the quasimolecular ion peak at *m/z* 823.2437 [M]^−^ (calcd. for C_41_H_43_O_18_, 823.2449) in the negative HR-ESI-MS, indicating 20 double bond equivalents. The IR (KBr) spectrum showed characteristic absorption bands for hydroxyl groups (3,440 cm^−1^), methylene groups (2,923 and 1,443 cm^−1^), aromatic rings (1,514 cm^−1^) and olefinic groups (1,630 and 986 cm^−1^). The ^1^H-NMR spectrum (Table 1) of compound **1** showed two sets of signals. The former set of signals, between *δ* = 6.0 and 7.5 ppm, was assigned to the protons of a *trans*-olefinic group (*δ* = 7.22 and 6.60 ppm, d, *J* = 16.0 Hz), two aromatic rings with 1,3,5-trisubstituted (*δ* = 7.06 ppm, d, *J* = 8.4 Hz; 6.90, d, *J* = 1.9 Hz and 6.82, dd, *J* = 8.4, 1.9 Hz) and 1,2,3,4-tetrasubstituted (*δ* = 6.51 ppm, d, *J* = 2.3 Hz; 6.28 ppm, d, *J* = 2.3 Hz) systems. The HMBC (Figure 2) correlations between *δ*_H_ 6.51 (H-6) with *δ*_C_ 120.2 (C-2), 102.8 (C-4), 156.6 (C-5) and 128.1 (C-α); *δ*_H_7.22 (H-α) with *δ*_C_ 140.1 (C-1), 120.2 (C-2), 105.0 (C-6), 135.4 (C-1') and 129.4 (C-β); *δ*_H_ 6.60 (Η-β) with *δ*_C_ 140.1 (C-1), 115.0 (C-2'), 120.0 (C-6'), 135.4 (C-1') and 128.1 (C-α); indicated the presence of a stilbene skeleton. The latter set of signals, between *δ* = 3.0 and 5.0 ppm, was assigned to the glycosyl protons and the methylene protons (*δ* = 4.11 ppm, s), consistent with the ^13^C-NMR spectrum along with the DEPT spectra of compound **1** (Table 1), which showed six signals characteristic of a glucosyl group (*δ* = 104.3, 74.9, 77.5, 71.3, 78.3 and 62.4 ppm) and a methylene carbon (*δ* = 21.3 ppm). The sugar residue was identified as a d-glucopyranosyl unit by gas chromatography of the hydrolyzed product. The mode of the glucosyl linkage was determined to be β from the coupling constant value (d, *J* = 7 Hz) of the anomeric proton signal. The location of the glucosyl group is suggested to be C-4' by HMBC, which displayed a correlation from *δ*_H_ 4.75 (H-1'') to *δ*_C_ 146.1 (C-4'). These moieties account for ten degrees of unsaturation, only half of those in the molecular formula of compound **1**. This result indicated that the structure of compound **1** was symmetrical. In summary, detailed analysis of 1D and 2D-NMR spectra suggested that the structural features of the symmetrical moieties of **1** was very similar to those of piceatannol-4'-*O*-β-d-glucopyranoside, which was previously isolated from *R.* *australe* as the main component, except for the presence of a CH_2_ group at position 2 (*δ* = 120.2 ppm). The connection of the symmetrical units was established by the HMBC experiment (Table 1), clearly indicating the correlation peaks from the methylene protons (*δ* = 4.11 ppm, s) to C-1 (*δ* = 140.1 ppm), C-2 (*δ* = 120.2 ppm) and C-3 (*δ* = 156.4 ppm), suggested that the connection group is the methylene at C-2. Therefore, the structure of compound **1** was established as shown in Figure 1. The ^1^H-NMR and ^13^C-NMR (100 MHz) spectral assignments performed by extensive 2D-NMR experiments (HSQC and HMBC) are summarized in Figure 2 and Table 1.

**Table 1:** ^1^H, ^13^C-NMR and HMBC (500M Hz) data of **1** (CD_3_OD, *δ* in ppm).

Position	*δ*_H_ (Mult., *J* in Hz)	*δ*_C_	DEPT	HMBC (Selected)
1		140.1	C	
2		120.2	C	
3		156.4	C	
4	6.28 (d, 2.3)	102.8	CH	C-2, 3, 5, 6
5		156.6	C	
6	6.51 (d, 2.4)	105.0	CH	C-2, 4, 5
1'		135.4	C	
2'	6.90 (d, 1.9)	115.0	CH	C-3', 4', 6'
3'		147.9	C	
4'		146.1	C	
5'	7.06 (d, 8.4)	118.5	CH	C-1', 3', 4'
6'	6.82 (dd, 8.5, 1.9)	120.0	CH	C-2′, 4′
α	7.22 (d, 16.0)	128.1	CH	C-1, 2, 6, 1'
β	6.60 (d, 16.0)	129.4	CH	C-1, 2', 6', 1'
1''	4.75 (d, 7.6)	104.3	CH	C-4'
2''	3.49 (m)	74.9	CH_2_	
3''	3.49(m)	77.5	CH_2_	
4''	3.42 (m)	71.3	CH_2_	
5''	3.42 (m)	78.3	CH_2_	
6''	3.91 (brd)	62.4	CH_2_	C-4'', 5''
	3.74 (dd, 11.4, 4.6)			
CH_2_	4.11 (s)	21.3	CH_2_	C-1, 2, 3

### 2.2. Antioxidant Activities by DPPH Scavenging Capacities

Aqueous and methanolic extracts of *R.* *australe* were reported to exhibit prominsing antioxidant activities in a previous study [16]. The most abundant stilbenoid of *R.* *austral*, piceatannol-4'-*O*-β-d-glucopyranoside, was suggested to be an important constituent responsible for the antioxidant potential of the extracts of plant materials collected from Tibet [7]. In order to evaluate the potentials of other types of phenolic constituents, compounds **1**–**10** were screened for their antioxidant activities by the DPPH free radical-scavenging assay that has been widely used for the evaluation of antioxidant activities of natural products. The results obtained in this study are summarized in Table 2. Among these compounds, rheumaustralin (**1**) displayed relatively strong antioxidant activity with an IC_50_ value of 2.3 µM, lower than piceatannol (IC_50_ = 0.14 μmol/L), and higher than resveratrol (IC_50_ = 15.6 μmol/L). This result was consistent with the concept that the antioxidant activity of stilbenoids depends on the position of the hydroxyl groups. The existence of *para*-hydroxyl groups significantly enhance antioxidant activity [17]. The glycosylation of hydroxyl groups, however, may decrease the antioxidant activity of stibenoid.

**Table 2:** Radical scavenging activities of the compounds **1**–**10**.

Compounds	DPPH Radical IC_50_ (μM) ^a^
**1**	2.3 ± 0.5
**2**	31.7 ± 1.1
**3**	25.7 ± 0.7
**4**	66.9 ± 1.3
**5**	21.7 ± 1.1
**6**	32.1 ± 1.5
**7**	56.4 ± 0.9
**8**	109.7 ± 2.1
**9**	69.7 ± 1.5
**10**	23.4 ± 0.8
resveratrol ^b^	15.6 ± 0.7
piceatannol ^b^	0.14 ± 0.05
ascorbic acid ^b^	19.7 ± 0.8
BHA ^b^	18.7 ± 0.9
α-tocopherol ^b^	25.1 ± 1.1

^a^ IC_50_ values were expressed as means ± standard deviation of three independent replicates; ^b^ Positive control substance.

Compounds **2**, **3**, **5**, **6** and **10** exhibited promising antioxidant activities with IC_50_ values in the range of 20 to 35 µM, which was comparable to those of BHA, ascorbic acid and α-tocopherol; while **4**, **7** and **9** showed moderate activities with IC_50_ values in the range of 50 to 70 µM. Compound **8** showed lowest activity with an IC_50_ value of 109.7 µM.

## 3. Experimental

### 3.1. General

The ^1^H-, ^13^C-, and 2D-NMR spectra were recorded on Bruker DRX-500 (500 MHz) spectrometer with TMS as internal standard. The ESI-MS and HR-ESI-MS spectra were recorded on VG AutoSpe 3000 and API Qstar P ulsar LC/TOF spectrometers, respectively. The UV spectra were measured by using a Shimadzu double-beam 210A spectrophotometer. The IR spectra were recorded on a Bio-Rad FTS-135 spectrometer, in KBr pellets. The optical rotations were measured by using a SEPA-3000 automatic digital polarimeter. The column chromatographic separations were performed on silica gel (200–300 mesh size; Qingdao Marine Chemical Inc., Qingdao, China), or Lichroprep RP-18 gel (40–63 µm mesh size; Merck, Darmstadt, Germany). The column fractions obtained were monitored by TLC, and spots were visualized by heating the silica gel plates after spraying with 15% H_2_SO_4_ in water. The TLC and PTLC separations were performed on silica gel Gf 254 pre-coated plates (Qingdao Marine Chemical Inc.). 2,2-Diphenyl-1-picrylhydrazyl radical (DPPH), hexamethyldisilazane and trimethyl-chlorosilane were purchased from TCI (Shanghai, China), Piceatannol and resveratrol were isolated from *R.* *australe* D. Don [18].

### 3.2. Plant Materials

The underground parts of *R. australe* were collected in August 2010 from Doilungdêqên County, Lhasa, China, and authenticated by Prof. Zheng-Dong Fang of Shangri-La Alpine Botanic Garden (Yunnan, China) and re-identified by co-author Dr. Qing-Song Yang. A voucher specimen (No. 2010080401) was deposited in the School of Chemistry and Biotechnology, Yunnan University of Nationalities, Yunnan, China.

### 3.3. Extraction and Isolation of the Compounds

The air-dried powder of the underground parts (1.2 kg) of *R. australe* were extracted exhaustively with 75% aqueous EtOH (25 L) at room temperature for 5 times. The EtOH extract was concentrated *in vacuo* to yield a semi-solid (338 g), which was suspended in water (1000 mL), and extracted successively with petroleum ether (3 × 1000 mL) and EtOAc (3 × 1000 mL). The EtOAc organic phase was concentrated to yield a residue (225 g), which was loaded on a silica gel (SiO_2_) column (4 kg) and eluted with CHCl_3_/MeOH gradient to give four fractions (1–4). Fraction 1 eluted with CHCl_3_ was partly subjected to repeated column chromatography (CC; SiO_2_; CHCl_3_/MeOH, 15:1) to afford **11** (125 mg), **12** (295 mg), **13** (255 mg) and **14** (235 mg). Fraction 2 eluted with CHCl_3_/MeOH (10:1) was subjected to repeated CC (SiO_2_; PE/acetone, 8:2) to afford **3** (25 mg), **5** (17 mg) **6** (155 mg) and **17** (16 mg). Fraction 3 eluted with CHCl_3_/MeOH (8:2) was subjected to repeated CC (SiO_2_; CHCl_3_/MeOH, 10:1–8:2) to afford **2** (995 mg), **10** (8 mg), **15** (595 mg) and **16** (418 mg). Fraction 4 eluted with CHCl_3_/MeOH (6:4) was subjected to repeated CC (SiO_2_; CHCl_3_/MeOH, 10:1–8:2) to afford three sub-fractions (F4a, F4b and F4c). Sub-fraction F4b was partly separated by PTLC (CHCl_3_/MeOH, 9:1) to afford **4** (27 mg) and **7** (15 mg). Sub-fraction F4c was subjected to repeated CC (SiO_2_; CHCl_3_/MeOH, 10:1–5:1) to afford **8** (30 mg) and a mixture containing **8** and **9**. This mixture was subjected to repeated CC on RP_18_ gel eluted by MeOH/water (58:42) to afford **8** (10 mg) and **9** (12 mg). The remaining aqueous phase was subjected to CC on D101 macroporous resin and eluted with 30% MeOH to afford four main fractions (A–D). Fraction B (18 g) was subjected to CC on Sephadex LH-20 using MeOH as the elution solvent to afford five sub-fractions (B1–B5). Sub-fractions B3 and B4 was further separated by repeated CC on RP C-18 and eluted by 25% MeOH to afford compound **1** (627 mg) and **18** (21 mg).

### 3.4. Acid Hydrolysis of Compound **1**

A solution of compound **1** (7 mg) in 5% aqueous sulfuric acid (2 mL) was heated in a water bath (80 °C) for 4 h. The solution, after cooling, was diluted with H_2_O (2 mL), neutralized with 5% NaOH solution and then extracted with EtOAc (5 × 3 mL). The aqueous layer was concentrated under a stream of nitrogen. The residue was then dissolved in anhydrous pyridine (0.8 mL), followed by the addition of trimethysilylation reagent hexamethyldisilazane/trimethylchlorosilane/pyridine (HMDS/TMCS/pyridine, 3:1:8). It was then stirred at 60 °C for an additional 30 min. The solution was analyzed by GC for sugar identification. GC analyses were performed using an Agilent 5890 instrument on an Agilent HP-1 column (0.25 mm, 30 m, i.d., 0.25 µm). Temperatures of both the injector and detector were 200 °C. A temperature gradient system was used for the oven, starting at 150 °C and increasing up to 250 °C at a rate of 8 °C/min. D-Glucose was confirmed by comparison with the retention time of an authentic standard.

### 3.5. Spectroscopic Data

*Rheumaustralin* (**1**). Yellow powder; 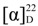 = + 50.3030^ο^ (*c* = 0.22, MeOH); IR (KBr) *ν*_max_ 3440, 2922, 1630, 1514, 1443, 1349, 1272, 1090, 986, 803 cm^−1^; UV (MeOH) *λ*_max_ (log *ε*) 223 (4.3), 325 (4.1) nm; positive ESI-MS [M+Na]^+^ at *m/z* 847; negative HR-ESI-MS [M−H]^−^ at *m/z* 823.2437 (calcd for C_4__1_H_43_O_18_ 823.2449); ^1^H- and ^13^C-NMR data (Table 1).

*Rheumin* (**2**). Yellow powder; EI-MS [M]^+^ at *m/z* 276; ^1^H-NMR (400 MHz, DMSO-*d*_6_) *δ* = 7.04 ppm (1H, d, *J* = 16.6 Hz, H-9), 6.92 (1H, d, *J* = 16.6 Hz, H-8), 6.46 (2H, d, *J* = 2.0 Hz, H-2', 6'), 6.24 (1H, d, *J* = 2.0 Hz, H-4'), 6.19 (1H, s), 5.63 (1H, d, *J* = 9.1 Hz, H-3), 2.72 (1H, dd, *J* = 2.5, 16.2 Hz, H-4), 2.18 (1H, dd, *J* = 9.3, 16.2 Hz, H-4); ^13^C-NMR (100 MHz, DMSO-*d*_6_) *δ* = 174.5 ppm (C-7), 172.7 (C-2), 158.8 (C-3', 5'), 138.9 (C-9), 137.1 (C-1'), 117.9 (C-8), 113.8 (C-6), 105.7 (C-2', 6'), 104.3 (C-4'), 80.6 (C-3) and 41.9 (C-4). These data are in accordance with those reported in the literature [19].

*2,5-Dimethyl-7-hydroxychromone* (**3**). Yellow needles, ^1^H-NMR (400 MHz, DMSO-*d*_6_) *δ* = 10.51 ppm (Ar-OH), 6.58 (1H, brs, H-8), 6.52 (1H, brs, H-6), 5.96 (1H, s, H-3), 2.59 (3H, s, 5-CH_3_), 2.25 (3H, s, 2-CH_3_); ^13^C-NMR (100 MHz, DMSO-*d*_6_) *δ* = 187.0 ppm (C-4), 165.7 (C-2), 163.7 (C-7), 159.2 (C-9), 141.0 (C-5), 118.5 (C-10), 111.1 (C-6), 101.7 (C-8), 98.5 (C-3), 22.9 (2-CH_3_), 14.8 (5-CH_3_). These data are in accordance with those reported in the literature [20].

*2,5-Dimethylchromone-7-O-β-D-glucopyranoside* (**4**). White powder, positive ESI-MS [M+Na]^+^ at *m/z*: 375; ^1^H-NMR (400 MHz, DMSO) *δ* = 6.85 ppm (1H, brs, H-8), 6.70 (1H, brs, H-6), 6.07 (1H, s, H-3), 4.63 (1H, s, anomeric H), 3.79–3.66 (2H, d, *J* = 10.4 Hz; H-6'), 3.32–3.13 (4H, t, *J* = 8.5 Hz; H-2'–H-5'), 2.73–2.67 (3H, d, *J* = 1.4 Hz, 5-CH_3_), 2.35–2.27 (3H, d, *J* = 1.6 Hz, 2-CH_3_); ^13^C-NMR (100 MHz, CDCl_3_) *δ* = 178.8 ppm (C-4), 164.9 (C-2), 160.4 (C-7), 159.3 (C-9), 141.8 (C-5), 117.1 (C-10), 116.6 (C-6), 111.5 (C-8), 101.9 (C-3), 100.3 (C-1'), 77.6 (C-2'), 76.9 (C-5'), 73.6 (C-3'), 70.1 (C-4'), 61.0 (C-6'), 22.8 (2-CH_3_), 19.9 (5-CH_3_). These data are in accordance with those reported in the literature [21].

*7-Hydroxy-2-(2'-hydroxypropyl)-5-methylchromone* (**5**). Yellow powder, positive ESI-MS [M+H]^+^ at *m/z*: 233; 1H-NMR (400 MHz, CDCl_3_) *δ* ppm 6.56 (1H, d, *J* = 2.0 Hz, H-8), 6.53 (1H, d, *J* = 2.0 Hz, H-6), 5.96 (1H, s, H-3), 4.10 (1H, m, H-2'), 2.62 (1H, dd, *J* = 5.1, 14.5 Hz, H-1'a), 2.62 (3H, s, 5-CH_3_), 2.55 (1H, dd, *J* = 8.0, 14.5 Hz, H-1'b), 1.18 (3H, d, *J* = 6.2 Hz, C-3'); ^13^C-NMR (100 MHz, CDCl_3_) *δ* = 182.0 ppm (C-4), 167.1 (C-9), 163.2 (C-7), 161.5 (C-2), 143.6 (C-5), 118.1 (C-3), 115.7 (C-10), 112.5 (C-6), 101.7 (C-8), 66.3 (C-2'), 44.2 (C-1'), 23.5 (C-3'), 23.2 (5-CH_3_). These data are in accordance with those reported in the literature [22].

*Torachrysone* (**6**). Yellow powder; positive FAB-MS [M+H]^+^ at *m/z* 247; ^1^H-NMR (500 MHz, CDCl_3_) *δ* = 7.09 ppm (1H, s, H-4), 6.83 (1H, d, *J* = 2.4 Hz, H-7), 6.78 (1H, d, *J* = 2.4 Hz, H-5), 3.78 (3H, s, OCH_3_), 2.38 (3H, s, COCH_3_), 2.17 (3H, s, CH_3_); ^13^C-NMR (125 MHz, CDCl_3_) *δ* = 204.7 ppm (COCH_3_), 158.1 (C-8), 154.7 (C-6), 150.8 (C-1), 134.9 (C-3), 133.8 (C-9), 122.6 (C-2), 118.1 (C-4), 107.9 (C-10), 102.9 (C-7), 102.5 (C-5), 55.8 (6-OCH_3_), 32.5 (COCH_3_), 19.8 (3-CH_3_). These data are in accordance with those reported in the literature [23].

*Torachrysone-8-O-β-D-glucopyranoside* (**7**). Yellow powder; positive FAB-MS [M+H]^+^ at *m/z* 409; ^1^H-NMR (500 MHz, DMSO-*d*_6_) *δ* = 9.47 ppm (1H, s, 1-OH), 7.12 (1H, s, H-4), 6.95 (1H, d, *J* = 2.5 Hz, H-7), 6.84 (1H, d, *J* = 2.5Hz, H-5), 5.12 (1H, d, *J* = 8.0 Hz, anomeric-H), 3.81 (3H, s, OCH_3_), 3.15–3.83 (6H, m, sugar-H), 2.48 (3H, s, COCH_3_), 2.21 (3H, s, CH_3_); ^13^C-NMR (125 MHz, DMSO-*d*_6_) *δ* = 205.1 ppm (COCH_3_), 158.3 (C-8), 155.4 (C-6), 151.2 (C-1), 136.9 (C-3), 133.7 (C-9), 123.2 (C-2), 118.8 (C-4), 108.7 (C-10), 103.1 (C-7), 102.7 (C-5), 101.2 (C-1'), 78.2 (C-5'), 76.7 (C-3'), 73.8 (C-2'), 70.4 (C-4'), 61.2 (C-6'), 55.8 (6-OCH_3_), 32.6 (COCH_3_), 20.1 (3-CH_3_). These data are in accordance with those reported in the literature [24].

*4-(4'-Hydroxyphenyl)-2-butanone-4'-O-β-D-glucopyranoside* (**8**). Colorless needles, negative ESI-MS [M−H]^−^ at *m/z* 325; 1H-NMR (400 MHz, CDCl_3_) *δ* ppm 7.14 (2H, d, *J* = 8.5 Hz; H-7, 9), 7.03 (2H, d, *J* = 8.5 Hz; H-6, 10), 4.88 (1H, d, *J* = 7.0 Hz, H-1'), 3.88–3.94 (2H, d, *J* = 10.8 Hz; H-6'), 3.80–3.40 (5H, m, H-2', 5'), 2.80 (4H, m, H-2, 3), 2.13 (3H, s, H-1). ^13^C-NMR (100 MHz, CDCl_3_) *δ* = 209.8 ppm (C-2), 156.1 (C-8), 135.0 (C-5), 128.9 (C-6,10), 116.4 (C-7,9), 101.1 (C-1'), 76.7 (C-3'), 76.6 (C-5'), 73.5 (C-2'), 70.0 (C-4'), 61.3 (C-6'), 44.6 (C-4), 25.6 (C-1, 3). These data are in accordance with those reported in the literature [25].

*Amabiloside* (**9**). Colorless needles, positive ESI-MS [M+H]^+^ at *m/z*: 300; ^1^H-NMR (400 MHz, CDCl_3_) *δ* = 9.79 ppm (1H, s, 1-CHO), 7.39 (1H, dd, *J* = 8.0, 2.0 Hz, H-6), 7.34 (1H, d, *J* = 2.0 Hz, H-2), 7.32 (1H, d, *J* = 8.0 Hz, H-5), 4.89 (1H, d, *J* = 7.6 Hz, H-1'), 3.55 (1H, dd, *J* = 9.5, 7.6 Hz, H-2'), 3.50 (1H, dd, *J* = 9.5, 7.6 Hz, C-3'), 3.48 (1H, m, C-5'), 3.41 (1H, dd, *J* = 9.5, 9.5 Hz, C-4'), 3.91 (1H, brd, *J* = 11.4 Hz, H-6'a), 3.74 (1H, dd, *J* = 11.4, 4.6 Hz, H-1'b); ^13^C-NMR (100 MHz, CDCl_3_) *δ* = 193.2 ppm (CHO), 152.4 (C-4), 149.0 (C-3), 133.4 (C-1), 125.0 (C-6), 117.1 (C-5), 116.3 (C-2), 102.8 (C-1'), 78.5 (C-3'), 77.5 (C-5'), 74.1 (C-2'), 71.2 (C-4'), 62.4 (C-6'). These data are in accordance with those reported in the literature [26].

*N-trans-feruloyl tyramine* (**10**). Amorphous solid, positive ESI-MS [M+Na]^+^ at *m/z*: 336; ^1^H-NMR (400 MHz, CD_3_OD) *δ* = 7.45 ppm (1H, d, *J* = 16.0 Hz), 7.14 (1H, d, *J* = 8.0 Hz), 7.08 (2H, d, *J* = 8.0 Hz), 7.03 (1H, d, *J* = 12.0 Hz), 6.81 (1H, dd, *J* = 8.0, 12.0 Hz), 6.74 (2H, d, *J* = 8.0 Hz), 6.43 (1H, d, *J* = 16 Hz), 3.88 (3H, s), 3.41 (2H, t, *J* = 8.0 Hz), 2.74 (2H, t, *J* = 8.0 Hz); ^13^C-NMR (100 MHz, CD_3_OD) *δ* = 169.1 ppm (C-1), 118.6 (C-2), 142.0 (C-3), 131.2 (C-1'), 130.7 (C-2',6'), 156.9 (C-4'), 116.2 (C-3',5'), 128.2 (C-1'''), 111.4 (C-2'''), 149.9 (C-3'''), 149.2 (C-4'''), 116.4 (C-5'''), 123.2 (C-6'''), 56.3 (OCH_3_), 42.5 (C-2''), 35.8 (C-3''). These data are in accordance with those reported in the literature [27].

### 3.6. DPPH Assays

The DPPH antioxidant assay was performed with slight modification from that reported previously [28]. Sample stock solution (1 mM) of rheumaustralin (**1**) was diluted to concentrations of 1.0, 3.0, 5.0, 7.0, 9.0 and 11.0 µM in methanol. Sample stock solution (10 µM) of piceatannol was diluted to concentrations of 0.05, 0.10, 0.15, 0.20, 0.25 and 0.30 µM in methanol.Sample stock solutions (1 mM) of resveratrol, ascorbic acid, butylated hydroxyanisole (BHA), (±)*-*α-tocopherol and compounds **2**–**10** were diluted to concentrations of 10, 30, 50, 70, 100 and 120 µM in MeOH. Two milliliter of DPPH methanol solution (100 µM, final concentration = 50 µM) was added to 2.0 mL of a methanol solution of various sample concentrations. The mixtures were shaken vigorously and then kept in dark at room temperature. After 30 min, the absorbance values were measured at 517 nm and converted into the percentage inhibition of DPPH (Ip) using the following formula:

Ip = [(Abs_sample_ − Abs_control_)/Abs_control_] × 100 (1)

A mixture of DPPH solution (2.0 mL, 100 µM) and methanol (2.0 mL) was used as the negative control.The IC_50_ values obtained represent the concentrations of the tested samples that caused 50% inhibition of DPPH radicals. The experiments were performed in triplicate, and the results are given as mean ± standard deviation (SD).

## 4. Conclusions

An unusual piceatannol dimer named rheumaustralin (**1**) was isolated from the underground parts of *R.* *australe* collected from Tibet, together with 17 known phenolic compounds **2**−**18**. Compounds **9** and **10** were isolated from *Rheum* plants for the first time. Stilbenoids such as resveratrol and piceatannol are widely distributed in higher plants as phytoalexins [29]. Some of their natural derivatives occur in oligomeric forms. The increasing degrees of polymerization and intriguing variety of polymerization patterns provide stilbene oligomers with dazzling chemical diversities. According to the patterns of oligomer construction and biosynthesis of stilbene oligomers reviewed recently [30,31], rheumaustralin (**1**), in which the stilbene units are connected only through a methylene (CH_2_) group, may represent a new connectivity pattern for these stilbene dimers. The discovery of this novel dimer further demonstrates the diversity of the stilbenoids from the genus *Rheum*. In addition, the free radical scavenging activities of compounds **1**–**10** against DPPH radicals have been evaluated in this study. All tested compounds showed bioactivites against DPPH radicals. Among them, rheumaustralin exhibited appreciable scavenging activity, with an IC_50_ value of 2.3 μM. Compounds **2**, **3**, **5**, **6** and **10** showed promising activities with IC_50_ values in the range of 20 to 35 µM, which was comparable to those of BHA, ascorbic acid and α-tocopherol. These phenolic compounds may have therapeutic potential and deserve further study.

## Supplementary Materials

Supplementary materials can be accessed at: http://www.mdpi.com/1420-3049/19/8/11453/s1..

## Supplementary Files

Supplementary File 1

## References

[1] Bao B., Alisa E.G., Li A., Bao B., Alisa E.G., Suk-pyo H., John M., Sergei L.M., Hideaki O., Chong-wook P. (2003). RHEUM Linnaeus. Flora of China.

[2] Xiao P., He L., Wang L. (1984). Ethnopharmacologic study of chinese rhubarb. J. Ethnopharmacol..

[3] Rokaya M.B., Münzbergová Z., Timsina B., Bhattarai K.R. (2012). *Rheum australe* D. Don: A review of its botany, ethnobotany, phytochemistry and pharmacology. J. Ethnopharmacol..

[4] Zargar B.A., Masoodi M.H., Ahmed B., Ganie S.A. (2011). Phytoconstituents and therapeutic uses of *Rheum emodi* wall. ex Meissn. Food Chem..

[5] Liu B., Yang J., Wang S. (2007). The chemical constituents in rhubarb rhizomes and roots derived from *Rheum emodi* Wall. Huaxi Yaoxue Zazhi.

[6] Wang A.Q., Li J.L., Li J.S. (2010). Chemical constituents of *Rheum emodi*. Zhong Cao Yao.

[7] Chai Y.Y., Wang F., Li Y.L., Liu K., Xu H. (2012). Antioxidant activities of stilbenoids from *Rheum emodi* Wall. Evid. Based Complement. Alternat. Med..

[8] Matsuda H., Morikawa T., Toguchida I., Park J.Y., Harima S., Yoshikawa M. (2001). Antioxidant constituents from rhubarb: Structural requirements of stilbenes for the activity and structures of two new anthraquinone glucosides. Bioorg. Med. Chem. Lett..

[9] Agarwal S.K., Singh S.S., Verma S., Kumar S. (2000). Antifungal activity of anthraquinone derivatives from *Rheum emodi*. J. Ethnopharmacol..

[10] Shi Y.Q., Fukai T., Sakagami H., Kuroda J., Miyaoka R., Tamura M., Nomura T. (2001). Cytotoxic and DNA damage-inducing activities of low molecular weight phenols from rhubarb. Anticancer Res..

[11] Suresh B.K., Tiwari A.K., Srinivas P.V., Ali A.Z., China R.B., Rao J.M. (2004). Yeast and mammalian α-glucosidase inhibitory constituents from Himalayan rhubarb *Rheum emodi* Wall ex Meisson. Bioorg. Med. Chem. Lett..

[12] Liang H.X., Dai H.Q., Fu H.A., Dong X.P., Adebayo A.H., Zhang L.X., Cheng Y.X. (2010). Bioactive compounds from *Rumex* plants. Phytochem. Lett..

[13] Xiang L., Lei F., Xing D., Wang W., Zheng J. (2005). Neuron protective constituents from *Rheum nanum* and *Rheum sublanceolatum*. Tsinghua Sci. Technol..

[14] Andersen D.O., Weber N.D., Wood S.G., Hughes B.G., Murray B.K., North J.A. (1991). *In vitro* virucidal activity of selected anthraquinones and anthraquinone derivatives. Antivir. Res..

[15] Liu W.B., Hu L., Hu Q., Chen N.N., Yang Q.S., Wang F.F. (2013). New resveratrol oligomer derivatives from the roots of *Rheum lhasaense*. Molecules.

[16] Rajkumar V., Guha G., Ashok Kumar R. (2011). Antioxidant and anti-cancer potentials of *Rheum emodi* rhizome extracts. Evid. Based Complement. Alternat. Med..

[17] Fang J.G., Lu M., Chen Z.H., Zhu H.H., Li Y., Yang L., Wu L.M., Liu Z.L. (2002). Antioxidant effects of resveratrol and its analogues against the free-radical-induced peroxidation of linoleic acid in micelles. Chem. Eur. J..

[18] Hu L., Chen N.N., Feng L., Hu Q., Liu W.B., Yang Q.S., Wang F.F. (2014). Piceatannol derivatives from *Rheum austral* D. Don and their chemotaxonomic significance. Biochem. Syst. Ecol..

[19] Li. J.L., Li. J.S., He. W.Y., Kong. M. (1998). Studies on the non-anthraquiones of *Rheum hotaoense*. Zhong Cao Yao.

[20] Kjer J., Wray V., Edrada-Ebel R., Ebel R., Pretsch A., Lin W., Proksch P. (2009). Xanalteric acids I and II and related phenolic compounds from an endophytic *Alternaria sp*. isolated from the mangrove plant *Sonneratia alba*. J. Nat. Prod..

[21] Zhao H.P., Wang Z.Y., Chen J.R., Li R.M., Wang Z.Q. (2009). New chromone glucoside from roots of *Rumex gmelini*. Nat. Prod. Res. Dev..

[22] Xu J., Kjer J., Sendker J., Wray V., Guan H., Edrada R., Proksch P. (2009). Chromones from the endophytic fungus *Pestalotiopsis* sp. isolated from the Chinese mangrove plant *Rhizophora mucronata*. J. Nat. Prod..

[23] Mei R., Liang H., Wang J., Zeng L., Lu Q., Cheng Y. (2009). New seco-anthraquinone glucosides from *Rumex nepalensis*. Planta Med..

[24] Demirezer Ö, Kuruüzüm A., Bergere I., Schiewe H.J., Zeeck A. (2001). Five naphthalene glycosides from the roots of *Rumex patientia*. Phytochemistry.

[25] Shikishima Y., Takaishi Y., Honda G., Ito M., Takeda Y., Kodzhimatov O.K., Ashurmetov O. (2001). Phenylbutanoids and stilbene derivatives of *Rheum maximowiczii*. Phytochemistry.

[26] Likhitwitayawuid K., Ruangrungsi N., Cordell G.A. (1993). Amabiloside, a new glycoside from *Crinum amabile*. Nat. Prod. Lett..

[27] Tanaka H., Nakamura T., Ichino K., Ito K. (1989). A phenolic amide from *Actinodaphne longifolia*. Phytochemistry.

[28] Sharma O.P., Bhat T.K. (2009). DPPH antioxidant assay revisited. Food Chem..

[29] Rivière C., Pawlus A.D., Mérillon J.M. (2012). Natural stilbenoids: Distribution in the plant kingdom and chemotaxonomic interest in Vitaceae. Nat. Prod. Rep..

[30] Xiao K., Zhang H.J., Xuan L.J., Zhang J., Xu Y.M., Bai D.L., Atta-ur-Rahman (2008). Stilbenoids: Chemistry and bioactivities. Studies in Natural Products Chemistry.

[31] Shen T., Wang X.N., Lou H.X. (2009). Natural stilbenes: An overview. Nat. Prod. Rep..

