# Supplementary material for: An Unusual Piceatannol Dimer from Rheum austral D. Don with Antioxidant Activity
Source: Molecules. 2014 Aug 4;19(8):11453–64. doi: 10.3390/molecules190811453 (PMC6271410; doi:10.3390/molecules190811453)

Supplementary Materials

Figure S1. ^{1}H-NMR spectrum (500M Hz, CD_3OD) of rheumaustralin.

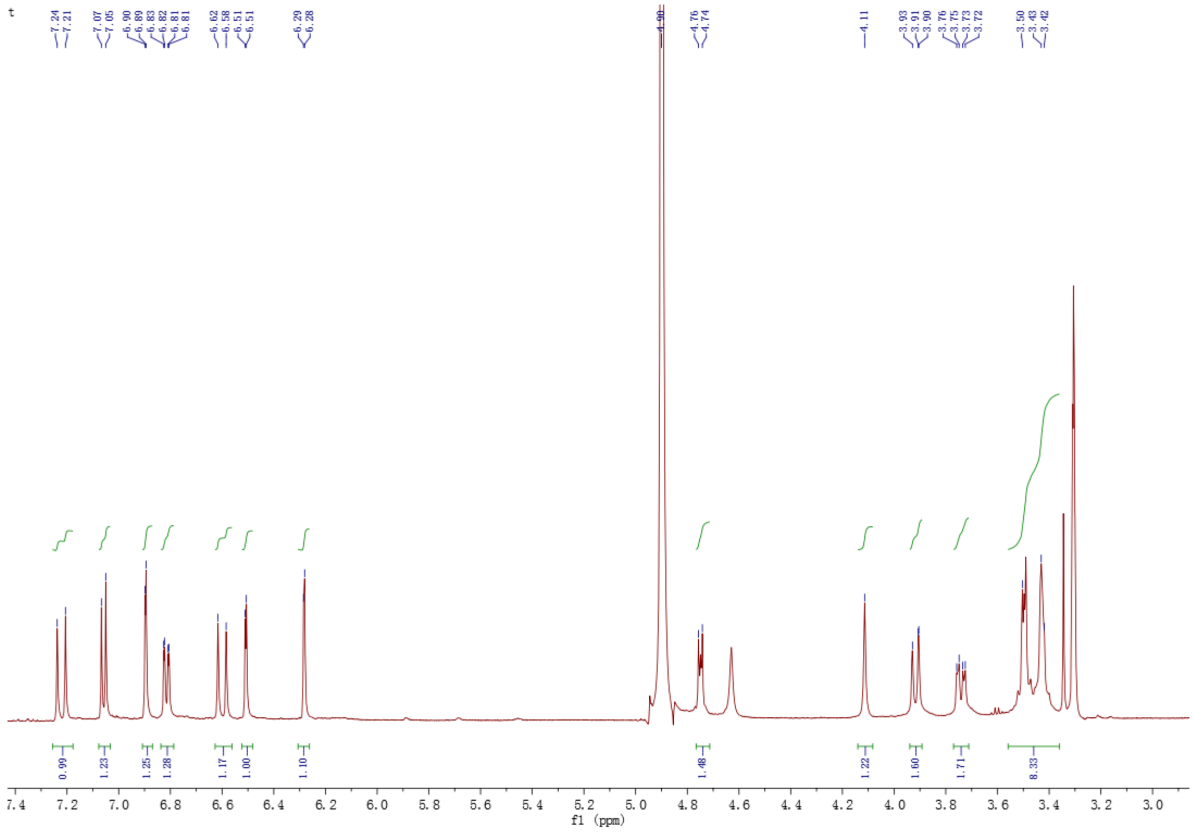

Figure S2. ^{13}C-NMR (DEPT) spectrum (125M Hz, CD_3OD) of rheumaustralin.

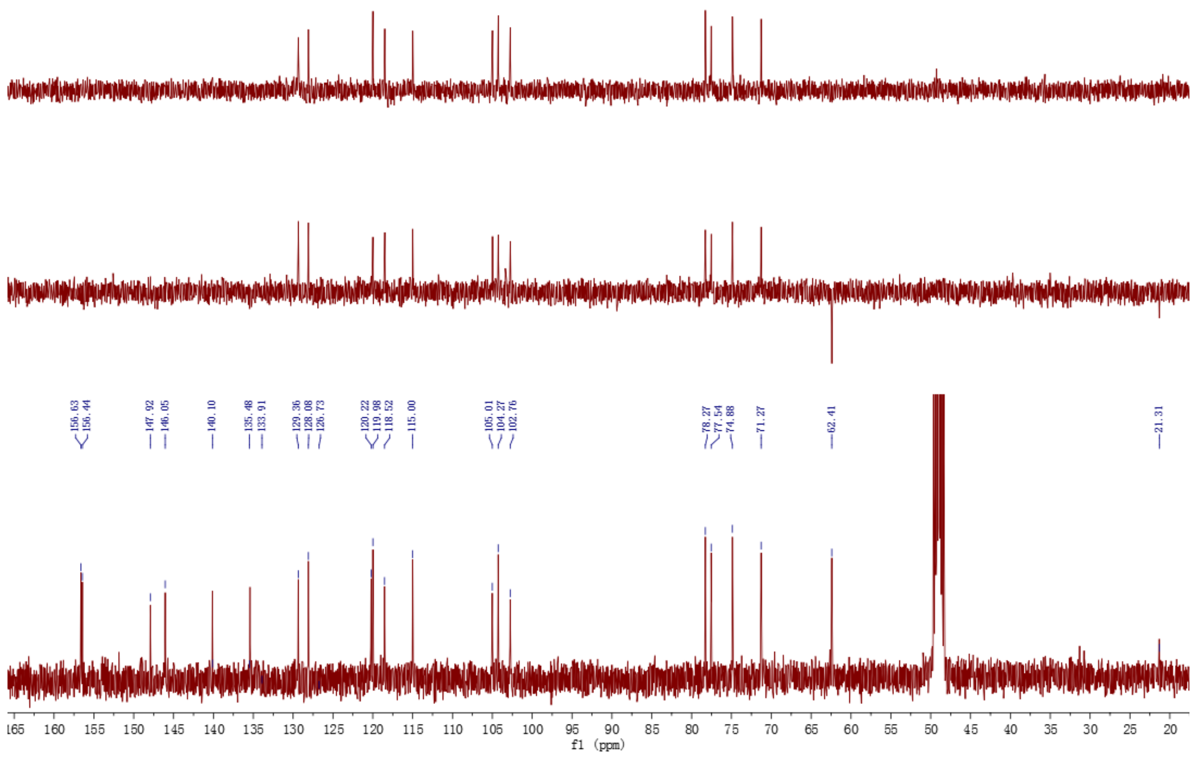

Figure S3. Detailed ^{13}C-NMR (DEPT) spectrum (δ ppm 170–110) of rheumaustralin.

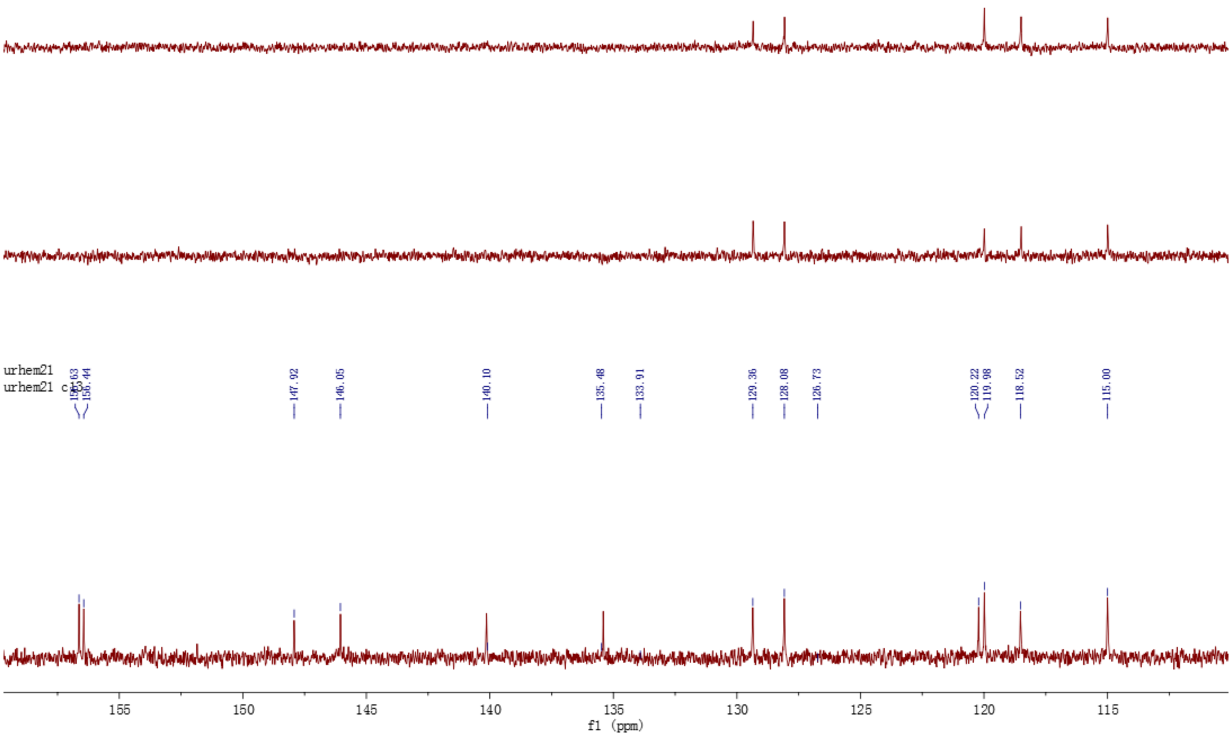

Figure S4. HSQC spectrum (500M Hz, CD_3OD) of rheumaustralin.

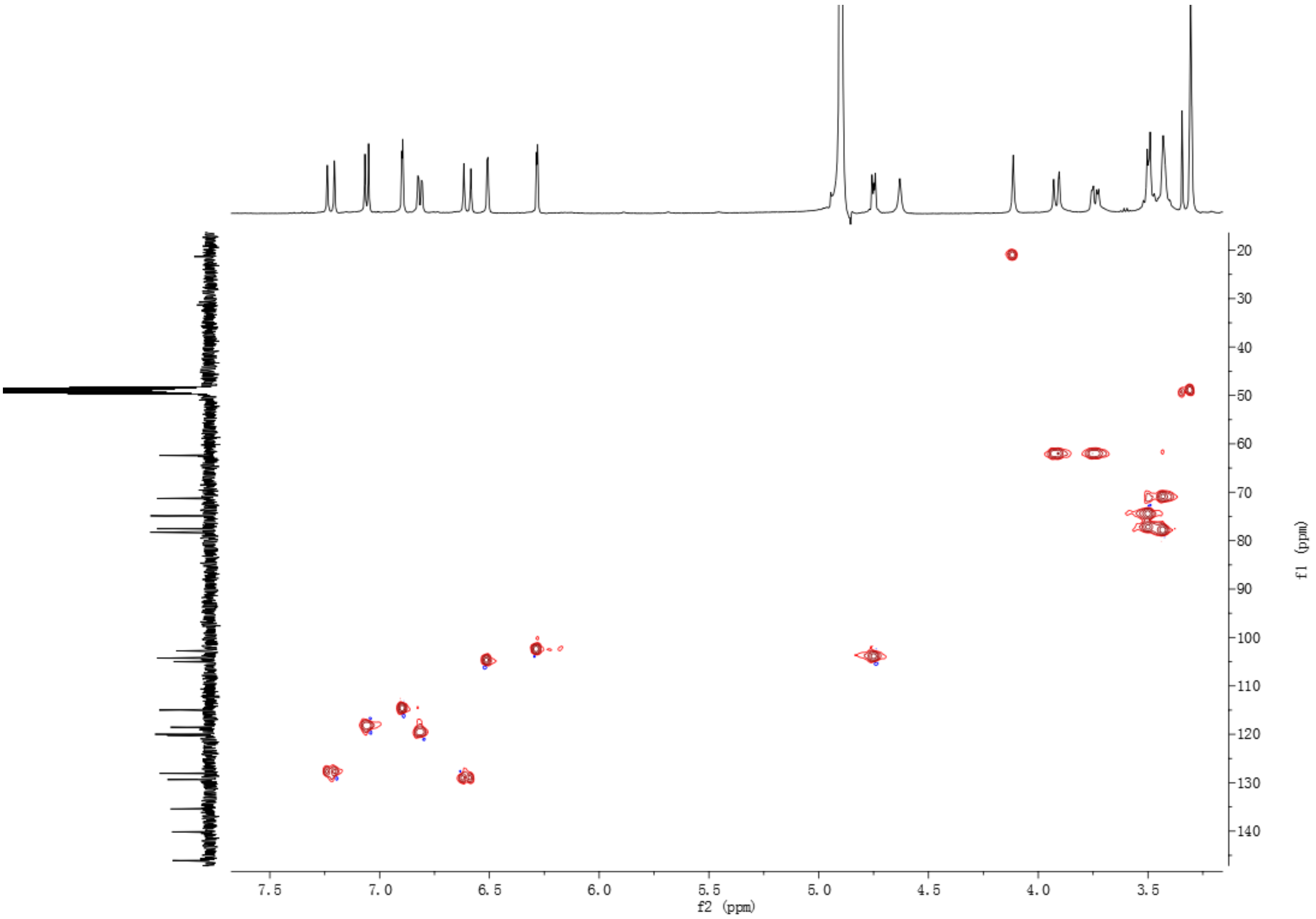

Figure S5. HMBC spectrum (500M Hz, CD_3OD) of rheumaustralin.

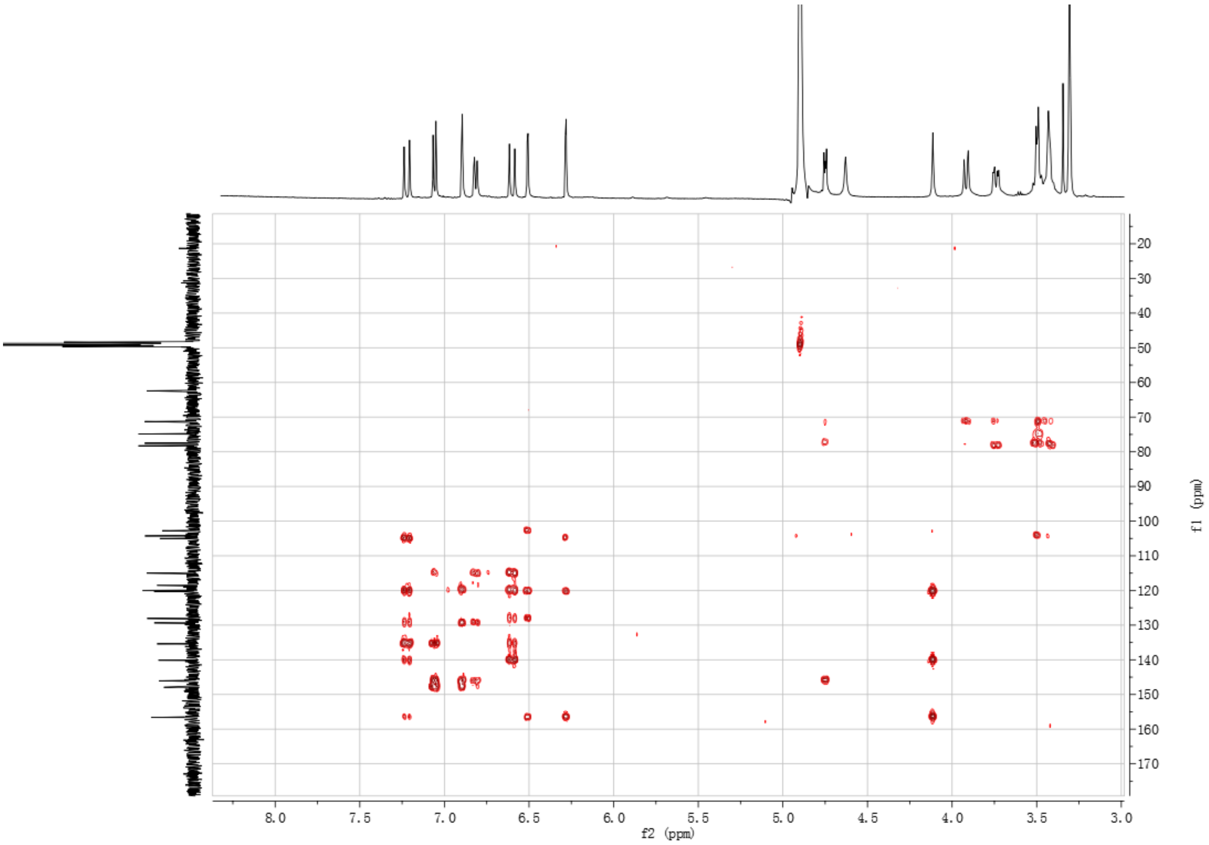

Figure S6. Detailed HMBC spectrum (δ_H ppm 4.00–6.16; δ_C ppm 165–110) of rheumaustralin.

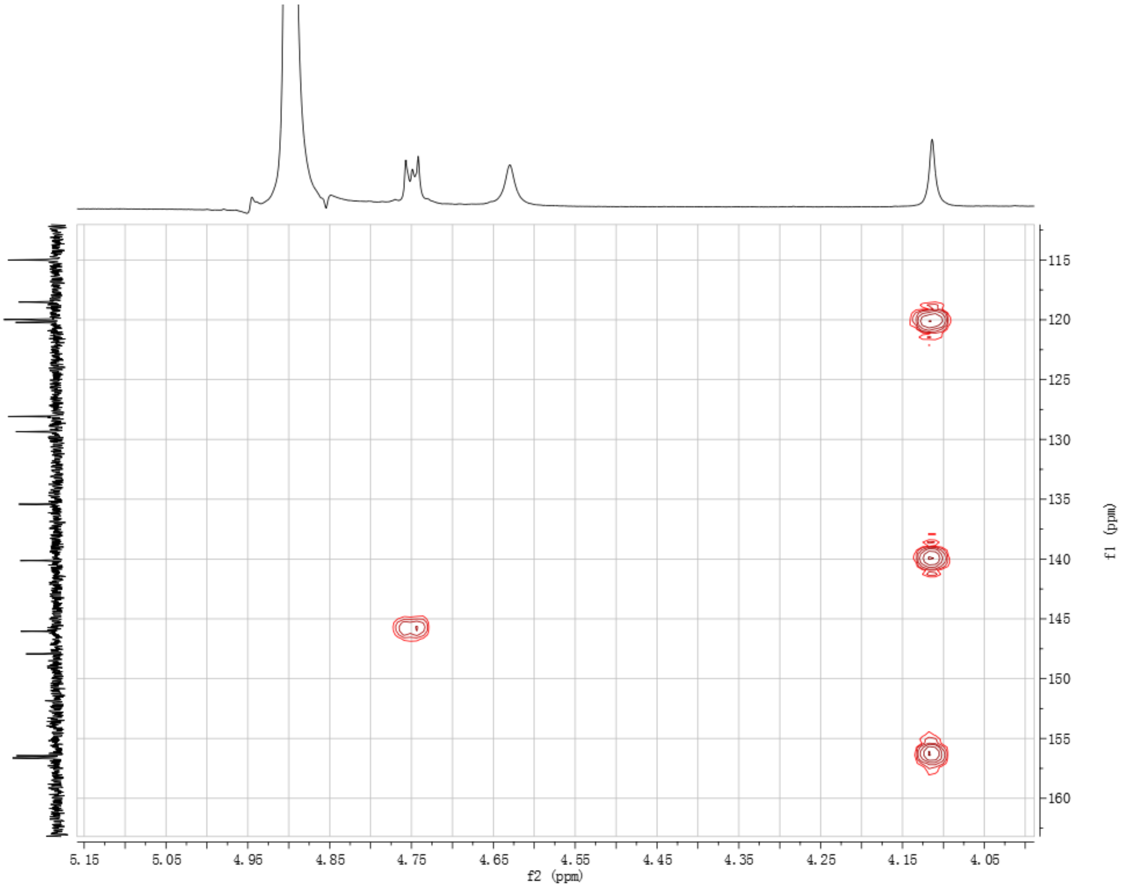

Supplement: Supplementary File 1 [file molecules-19-11453-s001.pdf]
